# Emerging Trends in Clinical Tropical Medicine Research

**DOI:** 10.4269/ajtmh.19-0043

**Published:** 2019-05-13

**Authors:** Mark K. Huntington, Joe P. Bryan, Troy D. Moon, Pascal J. Imperato, Susan L. F. McLellan, Walter R. Taylor, John S. Schieffelin

**Affiliations:** 1 Department of Family Medicine, University of South Dakota Sanford School of Medicine, Vermillion, South Dakota;; 2 Center for Global Health, Division for Global Health Protection, Centers for Disease Control and Prevention (retired), Atlanta, Georgia;; 3 Division of Pediatric Infectious Diseases, Vanderbilt Institute for Global Health, Nashville, Tennessee;; 4 SUNY Downstate School of Public Health, Brooklyn, New York;; 5 Division of Infectious Diseases, University of Texas Medical Branch, Galveston, Texas;; 6 Mahidol Oxford Research Unit, Bangkok, Thailand;; 7 Centre for Tropical Medicine and Global Health, University of Oxford, London, United Kingdom;; 8 Sections of Infectious Disease, Tulane University School of Medicine, New Orleans, Louisiana

## Abstract

The American Society for Tropical Medicine and Hygiene recently inaugurated an award for the best clinical research article published in the society’s journal in the previous year. This article summarizes both the process of selecting the winner and several themes that stood out in those articles which rose to the top for consideration. Themes of note included the importance of doing clinical research outside of referral centers, the complexity that must be considered when implementing interventions, incorporation of both ends of the age spectrum into studies, and considering cost-effectiveness and opportunity cost of interventions.

## INTRODUCTION

Historically, most clinical research projects have been focused on single disease–oriented topics and conducted in urban tertiary care centers. Clinical tropical medicine research efforts have largely followed suit. A developing trend in clinical research is to look beyond the traditional referral centers and their patient populations, out into the communities and to integrate the “horizontal” influences of comorbidities, culture, health delivery systems, social determinants of health, and other contexts, rather than maintain an artificially narrow “vertical” focus on single pathophysiological entities. This new approach, using community involvement and practice-based research networks, has the potential to challenge assumptions and uncover new strategies for improving the health of the individual and the community.^1^ In our review of clinical research articles in the journal over the past year, we found signs of a similar trend brewing in clinical tropical medicine research.

## BEST ARTICLE AWARD

In 2018, the American Committee on Clinical Tropical Medicine and Travelers’ Health (ACCTMTH), the American Society for Tropical Medicine and Hygiene’s clinical group, introduced an award to be given in recognition of the best clinical research article published in *The American Journal of Tropical Medicine and Hygiene* within the previous year. For the purposes of this award, clinical research was defined as the branch of health-care science that determines the safety and effectiveness of medications, devices, diagnostic products, and treatment regimens intended for human use.^2^ These may be used for prevention, diagnosis, treatment, or relieving symptoms of a disease. The scope also included studies of disease manifestation and progression, clinical epidemiological studies, and case reports of novel presentations or approaches to diagnosis or treatment. Review articles, meta-analyses, editorials, and treatment guidelines were excluded from consideration.

A selection committee of seven members was formed, composed of volunteers responding to the call for reviewers from members of ACCTMTH. All members of the committee were physicians, who have experience in both clinical care and research, as well as experiences working in global and/or low-resource settings. Each member reviewed, at minimum, a 3-month series of the journal to identify clinical research articles for consideration. A year’s worth of the journal, published between July 2017 and June 2018, was culled to 15 candidate articles in this first step (Table 1).

**Table 1:** Articles remaining following initial screening step, in alphabetical order by first author

Article	Topic	Summary
Boyce et al.^3*^†	Sign-disease association	About 1/5 of patients with fever in highland Uganda had parasitemia. Severe malaria manifested as shock and lactic acidosis much more commonly than severe anemia. Severe malaria had age peaks at 2–3 years and in those older than 50 years.
Bruxvoort et al.^6^	Effect of diagnostics on management	Rapid tests improved appropriate treatment with artemisinin combination therapy for test-positive and negative cases, but many cases were not treated according to test results.
Cash-Goldwater et al.^11^	Infection risk factors	Brucellosis confirmed by blood culture, or 4-fold rise in microagglutination was confirmed or probable in 9% of febrile patients, mostly who attend animals but were not suspected by medical providers.
Kakio et al.^12^	Medication quality	Studies of dissolution and use of handheld Raman spectrophotometer device were useful in detecting substandard or falsified medications using the antihypertensive candesartan as a test product.
Fauver et al.^13^	Xenosurveillance	PCR detected *Trypanosoma brucei gambiense*, *Bacillus anthracis*, Middle East respiratory syndrome–related Coronavirus, and Zika virus in the blood of *Anopheles gambiae* mosquitoes fed on blood containing these pathogens.
Huang et al.^14^	Outbreak	Relatives and a physician who cared for two index cases with this phlebovirus showed a spectrum of infection from asymptomatic to severe disease.
Hussain et al.^15^	Sanitation	Use of size-appropriate potties to catch and dispose of fecal material in children aged 6–36 months rather than open defecation resulted in improved sanitation. Acceptance varied with shape and color of the potties.
Lago et al.^8^†	Geriatric treatment	Individuals aged 60–85 years responded equally compared with persons aged 18–40 years but had increased cardiac arrhythmias and cardiac overload.
Lai et al.^16^	Diagnostics	*Plasmodium knowlesi* was detected within 20 minutes with high sensitivity and specificity.
Mitchell et al.^17^	Refugee health	Refugees in three Thailand–Burma border camps were screened and treated for intestinal parasites, evaluated for chronic hepatitis B, and screened and treated for anemia in addition to receiving appropriate vaccines. Results were forwarded to receiving health-care officials in the United States.
Morris et al.^7^	Water purification	Water quality was improved with the use of ceramic filters (< 1 *Escherichia coli*/100 mL). Visits for diarrhea were fewer in households using filters, but the incidence of diarrhea was not decreased. *Cryptosporidia* were not detected in large volumes of filtered water, and only 2% with ceramic filters.
Oldenburg et al.^9^†	Mass treatment	Treatment with azithromycin in 90% of persons in communities compared with 80% decreased infection (detected by PCR) more rapidly, but at 36 months, results were similar.
Pandey et al.^10^	Pediatric treatment	Administration of a single dose of liposomal amphotericin in 100 children with visceral leishmaniasis in India resulted in a cure rate of 100% at 1 month and 98% at 6 months. Fever and chills were side effects, but no nephrotoxicity was noted.
Poespoprodjo et al.^19^	Antimalarials	Once-daily treatment for 3 days resulted in clearance of 97.7% of *P. falciparum* and 98.2% of *P. vivax* cases by day 2. Day 42 efficacy was 97.7% and 98.2%, respectively. No genes associated with resistance were detected.
Refai et al.^18^	Non-pharmacologic treatment	Single 30-second heat treatment to 50°C was compared with treatment with intralesional stibogluconate in solitary lesions. Clinical response was faster in heat-treated lesions at 8 and 10 weeks, but similar thereafter. Heat treatment was well tolerated, less expensive, and painful than repeated intralesional injections.

* Ultimate selection.

† Three finalists.

At least two members of the selection committee reviewed each article identified, writing a summary and providing a score on a scale of 30. There were no a priori guidelines on how to score. Reviewers sought to identify those reports that demonstrated readability for a broad audience, and were both innovative and relevant with the potential to make a significant impact on health. The process resulted in three articles rising above the rest. These “finalists” were then ranked in order by each committee member, the synthesis of which resulted in selection of the article which would receive the honors,^3^ which were presented at the 2018 Annual Meeting.^4^

## NATURAL SELECTIONS

In addition to reports of clinical trials involving novel treatments or diagnostic technologies, the breadth of clinical studies now being published in the journal is impressive. This is reflected in articles that covered malaria treatment, its complications, and animal (now human) *Plasmodium knowlesi*; advances in the treatment of leishmaniasis; migration medicine; water, sanitation, and hygiene; disease elimination using mass drug treatment; One Health^5^ themes such as brucellosis; advances in xenodiagnoses; nosocomial spread of viral infections such as alpha virus in Asia causing severe fever and thrombocytopenia; and falsified medications. This represents a substantial increase in clinically applicable articles compared with a predominance of preclinical and basic science reports in the past.

Some interesting trends were noted in the articles that emerged through this process. One of the themes was the importance of doing clinical research outside of referral centers. A prime example of this was the finding that clinical characteristics of severe malaria presenting to rural, peripheral health centers were different from those previously observed in referral centers, and that there may be different methods that are optimal for the identification and management of severe malaria in these settings.^3^ This result—especially if replicated in similar settings—emphasizes the importance of conducting clinical and epidemiological research in the context of the primary care health system rather than only in larger centers, especially within narrow silos which can emerge from the way global health activities are funded by Western interests.

A related theme is the complexity which must be considered when implementing interventions. For example, one investigative team had a broad enough perspective to look beyond the narrow focus of the investigation protocol itself and identified unintended consequences of a well-conceived public health intervention.^6^ The reality of application may differ from the ideals of implementation because of complex interactions of many factors in the patient, the health system, and the community. As part of their discussion, they observed, “A more comprehensive approach to case management is needed, rather than focusing on only a single diagnosis and treatment.”^6^ This points to patient-oriented outcomes, rather than mere surrogate markers, as the true indicators of success. Patient-oriented outcomes are difficult to affect and measure because of the many factors in play. Another group included patient-oriented outcomes in the study design but ultimately emphasized a surrogate outcome when no significant effect was seen in the former.^7^

Another theme was the importance of incorporation of both ends of the age spectrum. The inclusion of geriatric populations—a group that is often neglected but is increasing in size in tropical clinical research—was refreshing.^8^ So, too, was the inclusion of the young.^9,10^ Whereas the findings may not have been surprising in terms of both efficacy and tolerability in children compared with young adults, toxicity in older patients with cutaneous leishmaniasis treated with systemic antimony drugs was increased to the point that it is not recommended.^8^ It was good to see actual data rather than leaving clinicians to extrapolate based on nonsimilar patient populations when making treatment decisions.

Finally, the importance of assessing cost-effectiveness and opportunity cost was apparent from the selected literature. Just because we can do something does not mean that we should—perhaps greater impact may be found elsewhere. One of the articles nicely described why diverting resources to extend the current 80% coverage of the intervention to 90% would not meaningfully decrease overall prevalence of the targeted disease. Instead, the resources could potentially make more significant health improvements if allocated to other areas.^9^

## SPONTANEOUS GENERATION

In selecting the “best clinical research article,” the committee was attempting to predict the future by identifying the work that will have the greatest effect on health in the tropics. The perspective reported here does not reflect a systematic review of the literature. It is the observation that those articles which most impressed the committee seemed to share these themes in common. The themes were not a priori selection criteria; they emerged from an ad hoc process affected by the shared biases of the selection committee. Ideally, these are biases which value recognition of a changing culture within clinical research, one that favors a more integrated and comprehensive approach to clinical tropical medicine research. We believe that the future will be shaped by well-designed studies that consider the entire community, including the complexity of interactions, the full span of its ages, and its priorities for health.
